# PLEUROPULMONARY HYDATID DISEASE TREATED WITH THORACOSCOPIC INSTILLATION OF HYPERTONIC SALINE

**DOI:** 10.4103/0970-2113.44138

**Published:** 2008

**Authors:** Hari Lakshmanan P, Monhammed Musthafa A, KP Suraj, C Ravidran

**Affiliations:** Institute of Chest Diseases, Medical College, Calicut., India

**Keywords:** Noninvasive Positive Pressure Ventilation, Hypercapnic Respiratory Failure

## Abstract

Hydatid disease is caused by the larval stage of the cestode, Echinococcus granulosus. Man is the intermediate host in its life cycle. The most common organ involved is liver followed by lung. Although surgery remains the definitive treatment for symptomatic lesions, it is associated with considerable morbidity. Other less invasive treatment strategies as an adjunct to medical treatment that have been tried in various case series include percutaneous aspiration, instillation and reaspiration of scolicidal agents (PAIR), and thoracoscopic removal of cysts located subpleurally. Here we report the case of a 58 year old gentleman with hepatic and pleuropulmonary hydatid disease who was subjected to medical thoracoscopy and instillation of hypertonic saline (3%), followed by medical management with albendazole with which complete resolution of the pulmonary cysts was achieved.

## CASE SUMMARY

**Fig. 1:**
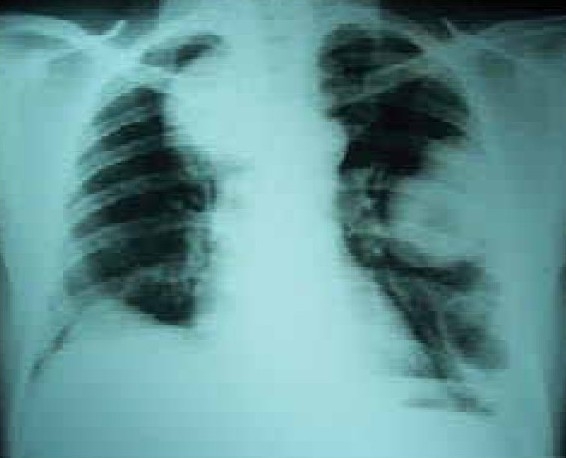
Chest X-Ray PA View (March '06)

**Fig.2a & 2b:**
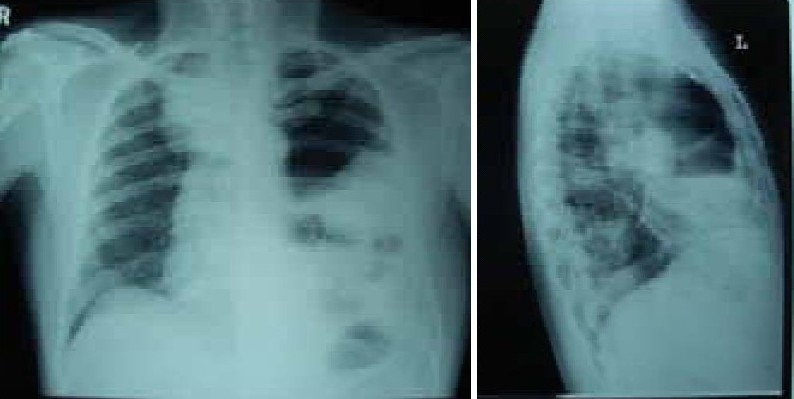
Fig. 2a Chest X-Ray PA View (May '06) Fig. 2b Chest X-Ray Left Lateral View (May '06)

**Fig. 3:**
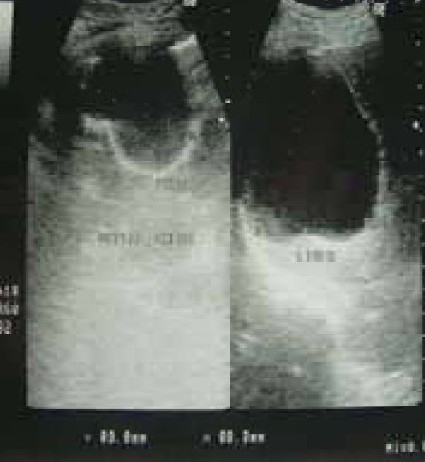
USG thorax and abdomen

A 58 year old male working in Saudi Arabia in an aquarium shop for more than 15 years was referred to our institute with complaints of breathlessness and vague abdominal discomfort of 8 months duration with worsening dyspnea and cough with scanty mucoid expectoration 1 month prior to admission. At his workplace, he had contact with aquatic creatures in the shop in which he was working. There was also occasional exposure to cats which was imported for sale and distribution to nearby places. The patient denied any addictions and there were no significant past medical or surgical illness. At the time of admission he was comfortable at rest, vitals were stable and respiratory system examination revealed features of left sided hydropneumothorax. Examination of other systems was within normal limits. Complete blood count, random blood sugar, renal and liver function tests were all normal. Chest X-ray taken at the time of presentation (Fig. 1) revealed a well defined mass lesion abutting the mediastinum in right upper zone, pneumothorax on left side with blunt costophrenic angle and large nodular lesions within the collapsed lung field on left side. Chest X-ray taken from the referral centre 2 months prior to the admission (Fig. 2a & 2b) showed 2 large well defined mass lesions one on each side with a cystic lesion in the left lower zone. An ultrasound examination of the thorax and abdomen (Fig. 3) had revealed multiple large lobulated cysts in liver and lung with two of them showing detached membranes. A CT Thorax (Fig. 4a–4d) was obtained which revealed left sided hydropneumothorax with multiple intrapulmonary and hepatic cysts suggestive of hydatid disease. A pleural aspiration was tried but did not yield any fluid. As the clinical and radiological features were suggestive of hydatid disease with rupture into the pleural cavity, we considered doing a thoracoscopy for this patient so as to visualize the cyst and to instill scolicidal agents into the cyst which is communicating with the pleura. Thoracoscopy was done under conscious sedation. The visceral pleural surface revealed remnants of a partially ruptured cyst and a glistening white membrane adherent to it (Fig. 5a,5b). Minimal amount of pleural fluid also was present. The remnants of ruptured cyst were removed, pleural fluid was sent for microbiological examination and pleural biopsy was obtained. 3% hypertonic saline was instilled into the pleural space as well as to the partially ruptured cyst; intercostal tube inserted and the incision was closed with tube insitu. A chest X-ray taken after the procedure showed clearance of the cystic shadow in left lower zone which was present in previous film. The pleural biopsy specimen did not reveal any microscopic evidence of hydatid larvae. The histopathology also was inconclusive. But as the clinical, radiological and thoracoscopic features were highly suggestive of hydatid disease, medical treatment with albendazole was continued along with praziquantel. Intercostal tube was removed after 10 days when there was complete lung expansion and negligible drain. The patient was subsequently discharged on albendazole and praziquantel. The patient came for follow-up after 2 months when he was asymptomatic. Follow-up X-ray showed compete clearance of the cysts on left side with only the right sided cyst remaining. As the patient was not willing for thoracotomy, and as the remaining lesions were asymptomatic, no further active interventions were made. Medical treatment was continued for 6 months. He remained asymptomatic on subsequent visits.

**Fig. 4a:**
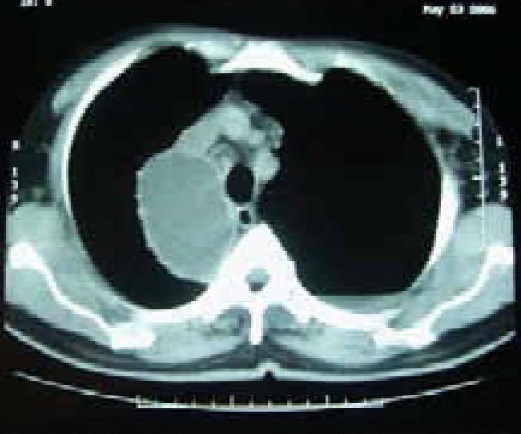
CT Thorax (May '06)

**Fig. 4b:**
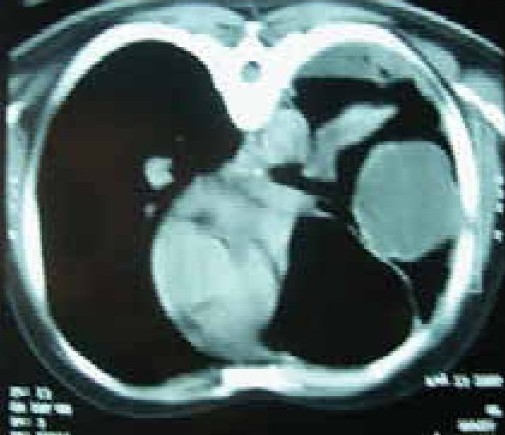
CT Thorax May '06)

**Fig. 4c:**
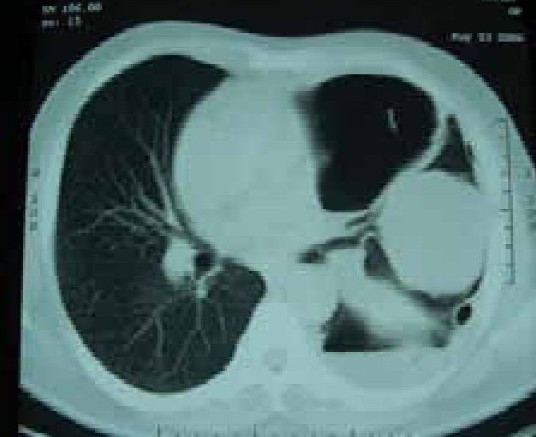
CT Thorax (May '06)

**Fig. 4d:**
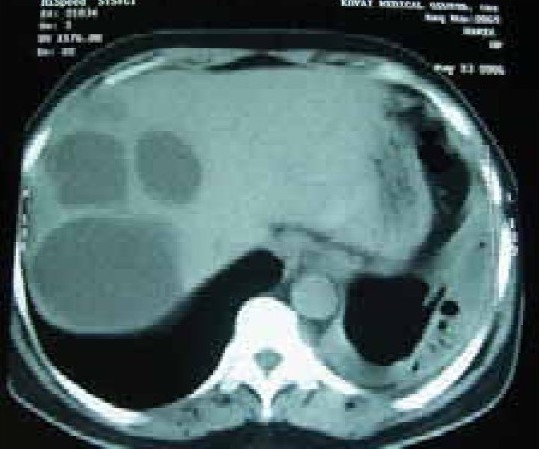
CT Liver (May '06)

**Fig. 5a:**
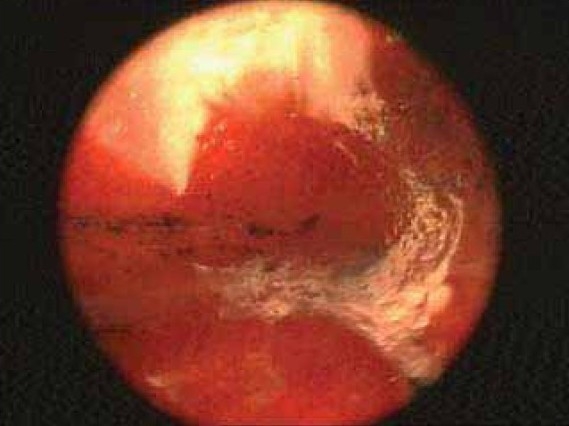
Thoracoscopy

**Fig. 5b:**
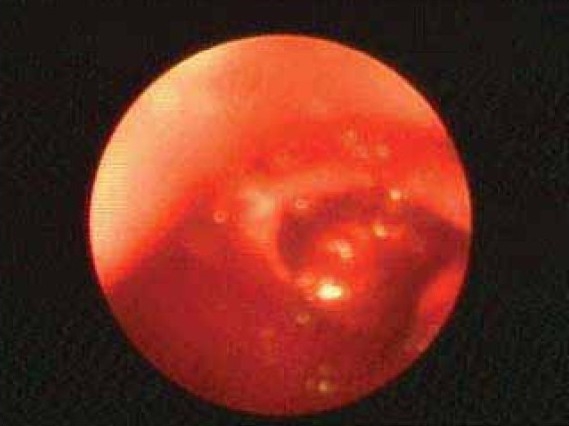
Thoracoscopy

**Fig. 6:**
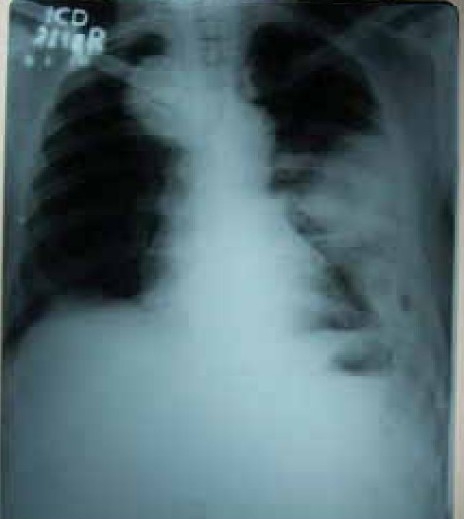
Chest X-ray post thoracoscopy

## DISCUSSION

Most of the case reports regarding hydatid lung disease published from India are from the northern states where it is relatively common. Similar case reports are scarce from southern regions. Also thoracoscopic instillation of scolicidal agents in the treatment is reported only in a few case series, although percutaneous aspiration, instillation and reaspiration (PAIR) is commonly employed for treating hepatic hydatid cysts. Sarai et al^1^ reported 16 cases of pulmonary hydatidosis which were treated with percutaneous instillation of hypertonic saline. They found early regression in all the cases even though subsequent regression was not seen. They noted better results with treatment of albendazole with or without surgey. Aribas et al^2^ reported the use of cystotomy with capitonage and decortication as the commonest treatment modalities employed in his series of 45 cases of hydatidosis presenting with pleural and pericardial complications. The morbidity and mortality rates were high in those undergoing radical surgical procedures for complicated cysts and the author concludes that such techniques should be better employed before the onset of complications. Islambekov et al^3^ also reports radical surgical techniques as the procedure of choice for hydatidosis that has ruptured into the pleural cavity. Thus even though radical surgical procedures remain the treatment of choice in complicated hydatidosis, they are associated with considerable morbidity and mortality which warrants the need for less invasive but effficacious procedures in treating this condition and thoracoscopy is perhaps one such technique. Kerekes et al^4^ reports the successful use of videothoracoscopy and cystectomy in the treatment of pulmonary hydatid. The hospital stay, morbidity as well as recurrence rates were low in their patients. A few other authors also report successful cure of the disease by thoracoscopy^5^. We have used medical thoracoscopy in this case which is less invasive and needs only local anaesthesia when compared to Video Assisted Thoracoscopic Surgery. This case report should be encouraging to clinicians towards using newer less invasive and relatively efficacious modalities like medical thoracoscopy in a disease which otherwise carries high morbidity and mortality especially in complicated cases. This is especially true in elderly and those with co-morbid illnesses in whom surgery carries high risk and is contraindicated. Percutaneous drainage of cysts is recommended only for hepatic hydatid cysts. Chemotherapy with benzimidazoles (Mebendazole and albendazole) with or without praziquantel achieves a cyst disappearance of 30%, partial response in another 30% and no response in 40%. It is effective in small cysts (<4 cm diameter), cysts with thin walls and in younger patients. It is indicated in patients who are high risks for surgery, in patients with multiple peritoneal cysts, to prevent secondary echinococcosis after spillage during surgery, and as a concomitant therapy with percutaneous drainage. Recent data show that uninterrupted drug therapy for 3-6 months has better efficacy with no increase in adverse effects.^6^,^7^ In our case, the management of the rest of the cysts in the thorax and liver remains controversial. As the right sided cyst appears deeply seated it may not be accessible to thoracoscopic removal. Also radical surgery is not advisable as there is diffuse involvement of liver as well. However, in our case, all the intrapulmonary cysts have disappeared with 6 months of treatment with albendazole. Some authors recommend medical treatment for asymptomatic cysts and intervene only if serial ultrasound images show progressive increase in size. But the fact that hydatid cysts grow at a very leisurely pace makes it difficult to convince patients with asymptomatic cysts to wait for months if not years to see if they are or are not at risk. It is true that in the end either the host or parasite must die. But the odds are heavily stacked against the latter; even though liver cysts take a long time before showing which way they will go. For unknown reasons, some cysts may stop growing at any time while others reach an enormous size and remain clinically silent. Still, if serial sonographic studies at six monthly intervals show definite enlargement of a particular cyst, that particular cyst bears watching and perhaps elective surgical removal.^8^

## CONCLUSION

The use of minimally invasive technique like medical thoracoscopy under conscious sedation in the management of pulmonary hydatid located in the subpleural region as well as those with pleural complications seems promising and needs to be promoted in those patients who refuse surgery or in whom surgery is contraindicated due to various reasons. However, it should be stated that the optimum management of asymptomatic cysts which are diffusely distributed in thorax (that are difficult to access by thoracoscopy) as well as in liver (which would require extensive surgical resection) remains controversial and poses challenge to the treating clinician. A trial of prolonged treatment with albendazole may be tried as in our case which could be fruitful in certain cases.
